# Adaptive Plasticity in Wild Field Cricket’s Acoustic Signaling

**DOI:** 10.1371/journal.pone.0069247

**Published:** 2013-07-23

**Authors:** Susan M. Bertram, Sarah J. Harrison, Ian R. Thomson, Lauren P. Fitzsimmons

**Affiliations:** 1 Department of Biology, Carleton University Ottawa, Ontario, Canada; Université Paris 13, France

## Abstract

Phenotypic plasticity can be adaptive when phenotypes are closely matched to changes in the environment. In crickets, rhythmic fluctuations in the biotic and abiotic environment regularly result in diel rhythms in density of sexually active individuals. Given that density strongly influences the intensity of sexual selection, we asked whether crickets exhibit plasticity in signaling behavior that aligns with these rhythmic fluctuations in the socio-sexual environment. We quantified the acoustic mate signaling behavior of wild-caught males of two cricket species, *Gryllus veletis* and *G. pennsylvanicus.* Crickets exhibited phenotypically plastic mate signaling behavior, with most males signaling more often and more attractively during the times of day when mating activity is highest in the wild. Most male *G. pennsylvanicus* chirped more often and louder, with shorter interpulse durations, pulse periods, chirp durations, and interchirp durations, and at slightly higher carrier frequencies during the time of the day that mating activity is highest in the wild. Similarly, most male *G. veletis* chirped more often, with more pulses per chirp, longer interpulse durations, pulse periods, and chirp durations, shorter interchirp durations, and at lower carrier frequencies during the time of peak mating activity in the wild. Among-male variation in signaling plasticity was high, with some males signaling in an apparently maladaptive manner. Body size explained some of the among-male variation in *G. pennsylvanicus* plasticity but not *G. veletis* plasticity. Overall, our findings suggest that crickets exhibit phenotypically plastic mate attraction signals that closely match the fluctuating socio-sexual context they experience.

## Introduction

Organisms exhibit phenotypic plasticity when individual genotypes produce different phenotypes in different environments [1]–[3]. Phenotypic plasticity is adaptive when phenotypes are matched to the environment and result in elevated fitness [1], [4]–[7]. Adaptive phenotypic plasticity can evolve provided that genetic variation in phenotypic traits is sufficient, plasticity is not constrained by pleiotropy or epistasis, and costs remain relatively low [6], [8]–[14]. Adaptive phenotypic plasticity is most likely to evolve when variable environments are predictable [15].

Fitness plays a key role in determining costs and benefits of phenotypic plasticity, making sexually selected traits ideal for use in plasticity studies [14] because males with the most exaggerated sexual traits usually have the highest fitness [16], [17]. Sexual traits also typically reflect condition [18] since only individuals in top condition should be able to bear the costs of advertising with the most exaggerated traits [19]. Sexually selected traits should, therefore, act as phenotypically plastic gauges of an individual’s condition [18], [20]–[23]. Several studies have revealed that sexually selected traits exhibit adaptive phenotypic plasticity. Adaptively plastic sexual traits can be fixed and irreversible at maturity or can be highly flexible and changeable throughout adulthood, changing as different environments are encountered [18], [24], [25]. For example, there are two male morphs in dung beetles (*Onthophagus taurus*), large males with elaborate weaponry that guard their mates, and small males with reduced weaponry that sneak copulate [26]. The social environment in which mothers are reared influences horn length in mate-guarding sons, although not in sneak copulating sons. Mothers reared with conspecifics produce sons with longer horns, while mothers reared in isolation produce sons with shorter horns [27]. In contrast, in the two-spotted field cricket (*Gryllus bimaculatus*), mating strategies are flexible. Males in the presence of rivals court females sooner, at higher rates, and transfer larger spermatophores than they do in control non-rival environments [28]. These examples reveal that changes in the socio-sexual environment can result in adaptively plastic mating strategies (reviewed by [29]).

Here we explore whether rhythmic changes in the socio-sexual environment result in adaptively plastic mate attraction behaviors in North American spring (*G. veletis*) and fall (*G. pennsylvanicus*) field crickets. Male crickets signal acoustically to attract females using long distance mate attraction signals (chirps). They raise their forewings and rub them together; each closing stroke produces a pulse of sound, and males concatenate pulses into chirps [30]. Much is known about cricket long distance mate attraction signaling and mating behavior at the population level. Cricket density often changes in a rhythmic pattern as a result of predictable fluctuations in the biotic (predator and parasite density) and abiotic (light and temperature levels) environment (summarized in Table S1). For example, mate signaling in male Texas field crickets, *G. texensis*, aligns positively with female mating activity, but negatively with parasitoid tachinid fly (*Ormia ochracea*) host-searching activity [31], [32]. Given that the density of conspecific rivals and potential mates determines the intensity of sexual selection [33]–[38], the fitness of any given phenotype should also exhibit temporal rhythms that align with population rhythms. Males may therefore exhibit adaptive phenotypic plasticity in their mate attraction signals to match the temporally fluctuating competitive context they encounter [39]–[41].

Tantalizing evidence suggests that male crickets exhibit socio-sexual dependent differences in their signaling plasticity throughout the day, and that condition limits this plasticity. Wild *G. campestris* females exhibit natural rhythmic fluctuations in their mating behavior, with most mating occurring in the afternoon and early evening (12∶00–21∶00 h) and little occurring in the night and morning (21∶00–12∶00; [37]). Jacot et al. [37] provided supplemental food to wild *G. campestris* and revealed that better fed males align their signaling activity with mate availability. During times when female mate-searching activity was naturally reduced in the wild, the signaling effort of food-supplemented males did not differ from that of control males. However, during times when female mate-searching activity was naturally elevated in the wild, the signaling effort of food-supplemented males was significantly elevated compared to unfed conspecifics (controls). Food-supplemented males exhibit plastic signaling behaviors in the wild, optimizing their signaling effort to maximize their potential for mating success [37]. This phenotypic plasticity is likely adaptive, as female *G. campestris* preferentially mate with males that signal most often [42].

Here we quantify variation in field cricket mate signaling plasticity at the species and individual level. At the species level, we examine the temporal signaling rhythms of two cricket species. We captured spring and fall field crickets as adults in their natural environments and asked whether, under controlled abiotic and biotic conditions, males exhibited the same rhythmic signaling patterns as previously described in the wild. We focused on spring and fall field crickets for two reasons. First, the signaling and mating diel rhythms of wild *G. pennsylvanicus* and *G. veletis* have been described. *Gryllus pennsylvanicus* males have high mate signaling activity throughout the night that peaks just before sunrise; signaling activity is much lower during the day [32]. Male *G. pennsylvanicus* signaling diel rhythms are synchronized with female *G. pennsylvanicus* mating activity diel rhythms, with most mating (88%) occurring at night and around sunrise in the wild (highest activity: 22∶00–09∶59; lowest activity: 10∶00–21∶59; [32]). Conversely, in *G. veletis* the highest signaling activity starts just before sunrise and continues for several hours following sunrise; signaling activity is much lower in the late afternoon and early evening but starts to gradually increase following sunset. Male *G. veletis* signaling diel rhythms are synchronized with female *G. veletis* mating activity diel rhythms, with most mating (81%) occurring during the day, peaking around sunrise and for several hours following it in the wild (highest activity: 04∶00–15∶59; lowest activity: 16∶00–03∶59; [32]). Second, the geographic distributions and mating seasons of *G. veletis* and *G. pennsylvanicus* overlap spatially and temporally. In Ontario, Canada, temporal overlap in breeding seasons occurs as *G. veletis* breeds from May to early July, while *G. pennsylvanicus* breeds from June through October [32]. Spatial overlap also occurs, with both species preferring grassy fields and rocky crevices (SMB, SJH, IRT, and LPF personal observations). Hybridization is lethal between *G. pennsylvanicus* and *G. veletis* [43]. The temporal (within a day) differences in signaling activity described above have the potential to act as a behavioral barrier, preventing the potential mating that could occur when breeding seasons overlap. We compare and contrast signaling effort of field-captured *G. pennsylvanicus* and *G. veletis* monitored in the laboratory to reports of their signaling behavior in the wild.

**Figure 1:**
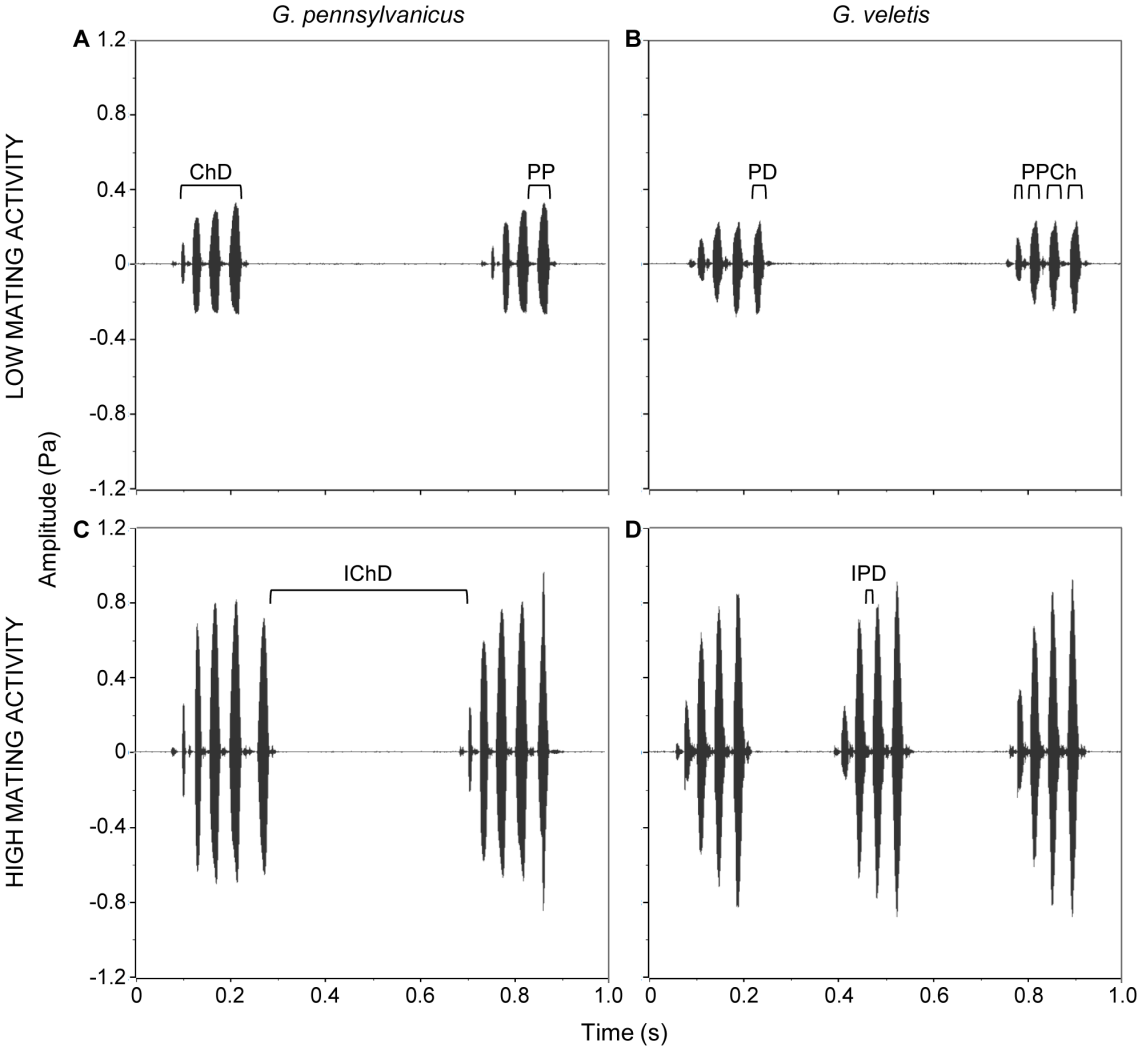
Waveforms of long-distance mate attraction signals of one *G. pennsylvanicus* and one *G. veletis* male. Figures show typical long-distance mate attraction signal for each species and how signaling typically changes during time periods indicative of low (A & B) and high (C & D) mating activity in the wild. Signal fine-scale properties are indicated as follows: ChD = chirp duration; IChD = interchirp duration; PPCh = pulses per chirp; PD = pulse duration; IPD = interpulse duration; and PP = pulse period, which combines PD and IPD.

We also provide the first species level investigation into how the fine scale structure of long distance mate attraction signals (Figure 1) changes over the course of the day. Plasticity in signal structure may be important because small differences in sound structure can influence female mating decisions. For example, females tend to prefer males that produce loud chirps (*G. lineaticeps*: [44]; *G. bimaculatus*: [45]) at low carrier frequencies (*G. bimaculatus*: [46]; *G. campestris*: [47], [48]; note *G. pennsylvanicus* prefer 5 kHz over 4 kHz signals [49]). Female crickets also prefer either average or elevated number of pulses per chirp (*G. campestris*: [46], [50]; *G. texensis*: [51]), species specific pulse periods (e.g. *G. campestris*: 40–60 msec [52]; *G. bimaculatus*: 40–60 msec [53]; *G. pennsylvanicus*: 35–60 msec [49], [54], *G. veletis*: 40–70 msec [54]), short chirp intervals (*G. integer*: [51]), high chirp rates (*G. lineaticeps*: [55]), long chirp durations (*G. lineaticeps*: [44]), and long calling bouts (*G. integer*: [56]). We therefore examine how *G. pennsylvanicus* and *G. veletis* alter the fine scale components of their signals through the course of the day.

At the individual level, we quantify phenotypic plasticity in mate attraction behavior. Given that the density of competitive rivals and female mates exhibit diel rhythms [32], the potential fitness payoffs of any given phenotype should also exhibit diel rhythms that align with species level rhythms. We therefore asked whether individual males exhibit phenotypic plasticity in their mate attraction signals that match the temporally fluctuating competitive and mating context they experience. We examined plasticity by comparing signaling behavior during two daily time periods: when mating activity is typically high in the wild versus when it is typically low. Higher mating activity means higher density of conspecific rival males and potential mates. We also examined whether body size (pronotum area) or residual mass (on body size) influenced signaling plasticity. We define condition as variation in resource acquisition and assimilation ability [57], which may result from differences in resource availability in the environment and/or individual physiological or genetic differences in the ability to locate, assimilate and utilize resources. Wild-caught crickets that naturally vary in body size and residual mass should allow us to explore the effect of natural variation in resource abundance and acquisition ability experienced during development in the wild. Bretman et al. [29] hypothesized males in high and low condition should exhibit minimal plasticity, whereas males in intermediate condition should display high plasticity, because intermediate condition males should have the stores available to amplify their signals when conditions are best but not enough stores to constantly maintain amplified signals. We therefore followed Bretman et al. [29] and predicted that males in average condition should display more adaptive signaling plasticity than males in high or low condition. Our correlational study exploring variation in plasticity is the first step towards determining whether crickets exhibit genotype by environment interactions in their signaling behaviors (eg. [58]–[60]).

## Methods

### Ethics Statement

Our study was conducted in accordance with the guidelines of the Canadian Council on Animal Care. We thank Art Weis and Koffler Scientific Reserve (Jokers Hill - University of Toronto) for allowing us to collect crickets on their property and for hosting our laboratory.

### Collection and Husbandry

We captured wild adult *Gryllus veletis* in Mississippi Mills, ON in May and June 2010, and *G. pennsylvanicus* at the Koffler Scientific Reserve (Jokers Hill - University of Toronto) near King City, ON in August 2010 (no collecting permits required). Crickets were housed individually in 520 mL clear plastic containers with a screened lid and crumpled unbleached paper towel as shelter. They were provided with unlimited water and food (powdered Harlan Teklad Inc. Rodent diet no. 8604 M, Harlan Laboratories, Madison, WI, USA). Crickets were transferred to Carleton University where they were housed in a temperature and photoperiod controlled greenhouse at 28±2°C on a 14∶10 h light:dark cycle.

### Acoustic Recording

We recorded the long distance mate attraction signals of wild-caught adult male *G. veletis* and *G. pennsylvanicus* by placing each male’s container in an electronic acoustic recording system (EARS-II; designed and developed for our laboratory by Cambridge Electronic Design, Cambridge, UK). *Gryllus veletis* males were caught locally so their acoustic mate attraction signals could be recorded in the EARS-II starting the morning immediately following capture. Following capture, *G. pennsylvanicus* had a 2–6 day delay before they could be recorded, resulting from our having to complete all field collections prior to transporting the crickets back to Carleton University. All males had their acoustic mate attraction signals recorded for 2–4 days in the EARS-II. We recorded a total of 63 *G. pennsylvanicus* and 32 *G. veletis*. Of these, almost all males signaled for mates, with the exception of 1 *G. pennsylvanicus* and 3 *G. veletis*.

The EARS-II consists of three units, each capable of recording the acoustic mate attraction signals of 32 males in real time (96 males in total; refer to [61] for detailed descriptions). Microphones (electret condenser type KECG2742PBL-A; Kingstate Electronics, Tamshui, Taipei, Taiwan) are positioned next to LED lights which are set to a 14∶10 h light:dark cycle. Each microphone and light combination is held 6.6-cm above the top of the male’s container. Males are separated from their neighbors by acoustically isolated enclosures (7-cm thick Styrofoam box lined with 3.5-cm thick acoustic foam) that contain both microphone and LED light. This design minimizes the likelihood of individuals detecting their neighbors’ signals. The Styrofoam boxes are configured in a 4×4 array on six shelving units within our greenhouse.

We recorded the long distance mate attraction calls of each male for 2–4 days. Sounds were recorded at 31.25 kHz. The microphones are continuously monitored and analyzed using CricketSong software (designed by Cambridge Electronic Design Ltd., Cambridge, UK; for details see [61]). The EARS II CricketSong software automatically filters out background noise and auto-adjusts the amplitude threshold for quiet or loud individuals. CricketSong software creates an acoustic file for each male for every hour he spends in the EARS II unit. We analyzed each acoustic file using Spike2 v6.12 (Cambridge Electronic Design, Cambridge, UK) to produce an hourly summary of the nine signaling parameters quantified: signaling time (# min in the hour), pulse duration (ms), interpulse duration (ms), pulse period (ms), pulses per chirp, chirp duration (ms), interchirp duration (ms), amplitude (dB), and carrier frequency (Hz) (Figure 1). Note, signaling time represents the total amount of time the individual spent signaling in that hour, whereas the other eight signaling parameters are represented by mean parameter values for that hour. We included pulse period, even though it is not a separate trait but instead combines pulse duration and interpulse duration, because females are known to prefer species specific pulse periods (*G. pennsylvanicus*: 35–60 msec [49], [54], *G. veletis*: 40–70 msec [54]). We included pulse duration and interpulse duration so that we could know what component of the signal males were changing to change pulse period.

### Body Size and Weight Measurements

Following acoustic recording we photographed live crickets in a dorsal position using a Zeiss Discovery V12 stereo dissecting microscope (AxioVision v4.8, Carl Zeiss; magnification: 5×, resolution: ∼1.60 µm). We used these photographs to quantify each male’s pronotum area (mm^2^). Males were weighed using a Denver Instruments balance (Pinnacle Series model PI-314; precision ±0.1 mg). Males were then added to the stock population to mate with collected females and build a laboratory culture.

### Statistical Methods

We performed statistical analyses in JMP v10.0.0 (SAS Institute Incorporated, Cary, NC, USA). Residual mass was quantified using a logistic regression of mass on pronotum area. Males were categorized as being in high, average, or low condition depending on whether they fell within the upper, middle, or bottom third of rank ordered residual mass scores. Similarly, males were categorized as high, average, or low condition depending on their rank ordered body size (pronotum area) scores.

We ensured that all parameters were normally distributed using Shapiro-Wilk Goodness of Fit tests. All parameters were normally distributed except signaling time and inter-chirp duration; we Box-Cox transformed these two parameters for both species. To quantify species-level differences in signaling behavior we used ANOVA. We compared nine different signaling parameters, and we corrected for multiple tests using Benjamini and Yekutieli’s [62] false discovery rate (FDR_B-Y_) method; our FDR_B-Y_ corrected alpha was P<0.0177. To visualize temporal signaling rhythms at the species level we quantified signaling behavior across 24 hours using six different time periods (period 1 = 00∶00–03∶59; 2 = 04∶00–07∶59; 3 = 08∶00–11∶59; 4 = 12∶00–15∶59; 5 = 16∶00–19∶59; 6 = 20∶00–23∶59). We visually compared our temporal rhythm plots that quantified wild-caught crickets signaling in the laboratory to previously published temporal rhythm plots that quantified wild-crickets signaling over a 24-hour period [32].

To investigate phenotypic plasticity in signaling behavior at the individual level we employed repeated measures general linear mixed models (GLMM) using restricted maximum likelihood (REML). We used the restricted maximum likelihood approach because we had missing cells (some males did not signal in a period on some days). The models included individual as a random effect, two time periods (high and low mating activity, see below for details) as a fixed effect, male condition (body size and residual mass) as fixed effects, and mating activity * residual mass and mating activity * body size interaction terms. Each male’s signals were repeatedly measured across the two mating activity levels previously defined by French and Cade [32] for wild populations: *G. pennsylvanicus* high = 22∶00–09∶59, low = 10∶00–21∶59; *G. veletis* high = 04∶00–15∶59, low = 16∶00–03∶59. We ran each of these models twice, once using continuous condition measures and once using categorical (high, medium, or low) condition variables. Our overall findings did not differ between the two model types (continuous or categorical condition measures), so we only present our categorical findings. We ran 36 different mixed effect models: 9 parameters×2 species×2 condition runs, and our FDR_B-Y_ corrected alpha level of significance was P<0.0120.

## Results

### Species Differences

**Figure 2:**
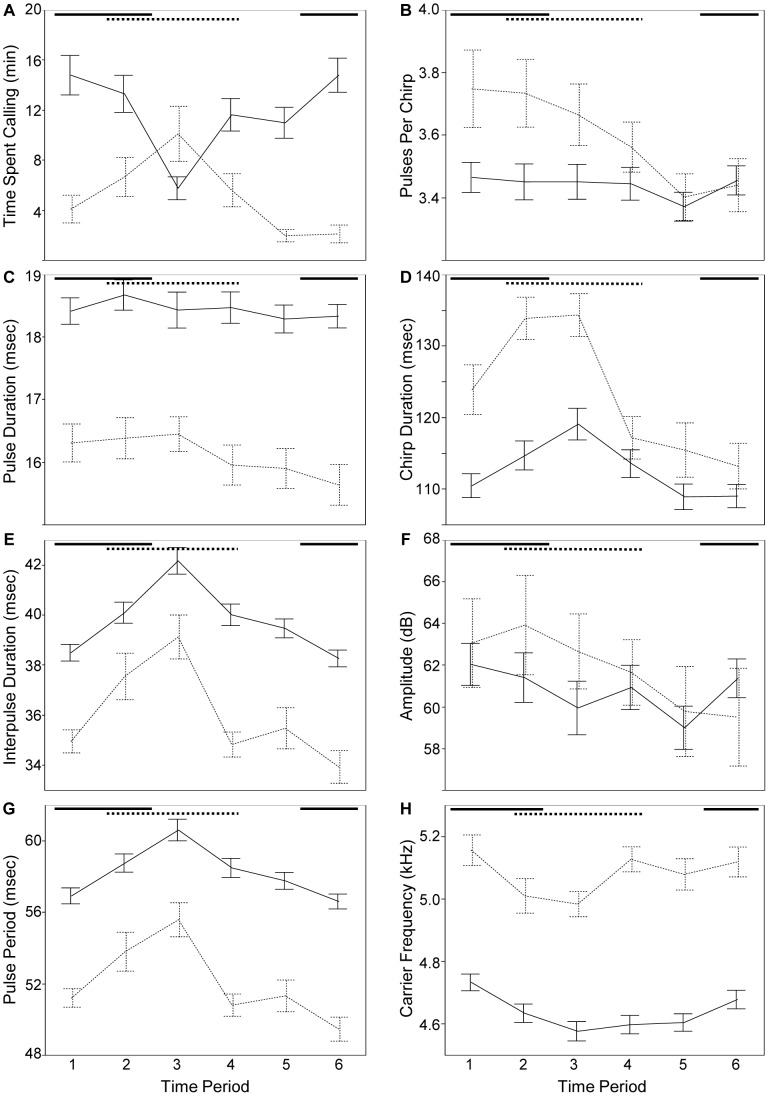
Signaling diel rhythms in *G. pennsylvanicus* (solid lines) and *G. veletis* (dotted lines). Error bars are standard error of the mean. Horizontal lines at the top of each panel depict the time of day that mating activity is highest (from [32]). Time periods: 1 = 00∶00–03∶59; 2 = 04∶00–07∶59; 3 = 08∶00–11∶59; 4 = 12∶00–15∶59; 5 = 16∶00–19∶59; 6 = 20∶00–23∶59.

Male crickets exhibited strong temporal rhythms in their signaling behavior that differed across species (Figure 2). During times of the day that *G. pennsylvanicus* mating activity is highest in the wild (22∶00–09∶59; [32]) males chirped more often and louder, with shorter interpulse, pulse period, chirp, and interchirp durations, and at slightly higher carrier frequencies. During times of the day that *G. veletis* mating activity is highest in the wild (04∶00–15∶59; [32]) males chirped more often, with more pulses per chirp, longer interpulse, pulse period, and chirp durations, shorter interchirp durations, and at lower carrier frequencies. Note: time effects were significant (Table S2). *Gryllus pennsylvanicus* and *G. veletis* also differed in other ways, with *G. pennsylvanicus* males signaling more often, at slightly lower carrier frequencies, with longer pulse, interpulse, pulse period, and interchirp durations, and shorter chirp durations than *G. veletis* (Table 1; Figure 2). Note, however, that there was extensive overlap between species in all traits. Both species chirped at similar amplitudes and included 3–4 pulses in their chirps (Table 1; Figure 2F and 2B, respectively), and kept their pulse durations relatively constant through time (Figure 2C).

**Table 1:** Interspecific differences in *G. pennsylvanicus* and *G. veletis* long distance mate attraction signaling parameters.

Signaling Trait	Species	Mean ± SD	Range	F	P	DF	R^2^_adj_
Signaling Time	*G. pennsylvanicus*	12.11±8.05	0–34.78	59.1074	**<0.0001**	1,93	0.3820
(min/hour)	*G. veletis*	5.18±5.56	0–19.73				
Pulse Duration	*G. pennsylvanicus*	18.42±1.55	14.61–22.43	46.8997	**<0.0001**	1,89	0.3377
(msec)	*G. veletis*	16.13±1.35	13.64–17.94				
Interpulse Duration	*G. pennsylvanicus*	39.31±2.54	33.99–44.81	14.5101	**0.0003**	1,89	0.1305
(msec)	*G. veletis*	36.89±3.38	32.81–45.43				
Pulse Period	*G. pennsylvanicus*	57.74±3.20	49.22–66.45	37.2571	**<0.0001**	1,89	0.2872
(msec)	*G. veletis*	53.02±3.89	47.65–63.37				
Pulses Per Chirp	*G. pennsylvanicus*	3.46±0.35	2.53–4.04	2.9071	0.0917	1,89	0.0208
(count)	*G. veletis*	3.60±0.43	2.71–4.67				
Chirp Duration	*G. pennsylvanicus*	112.02±12.74	75.89–132.66	23.2040	**<0.0001**	1,89	0.1979
(msec)	*G. veletis*	126.14±13.63	101.06–151.91				
Interchirp Duration	*G. pennsylvanicus*	918.27±278.07	442.52–1774.00	18.9743	**<0.0001**	1,89	0.7996
(msec)	*G. veletis*	564.64±137.06	371.85–852.20				
Amplitude	*G. pennsylvanicus*	61.01±7.30	41.06–81.72	0.2757	0.6009	1,89	0.0081
(dB)	*G. veletis*	61.88±7.59	47.25–79.03				
Carrier Frequency	*G. pennsylvanicus*	4.64±0.21	4.16–5.11	78.6988	**<0.0001**	1,89	0.4633
(kHz)	*G. veletis*	5.05±0.20	4.63–5.43				

SD = standard deviation; F = equality of variances test; Our corrected alpha FDR_B–Y_ level of significance is P<0.0177 to account for the 9 models run; DF = degrees of freedom; R^2^_adj_ = ratio of variability between group means to the overall sample variability, adjusted for number of explanatory terms.

### Individual Differences

**Figure 3:**
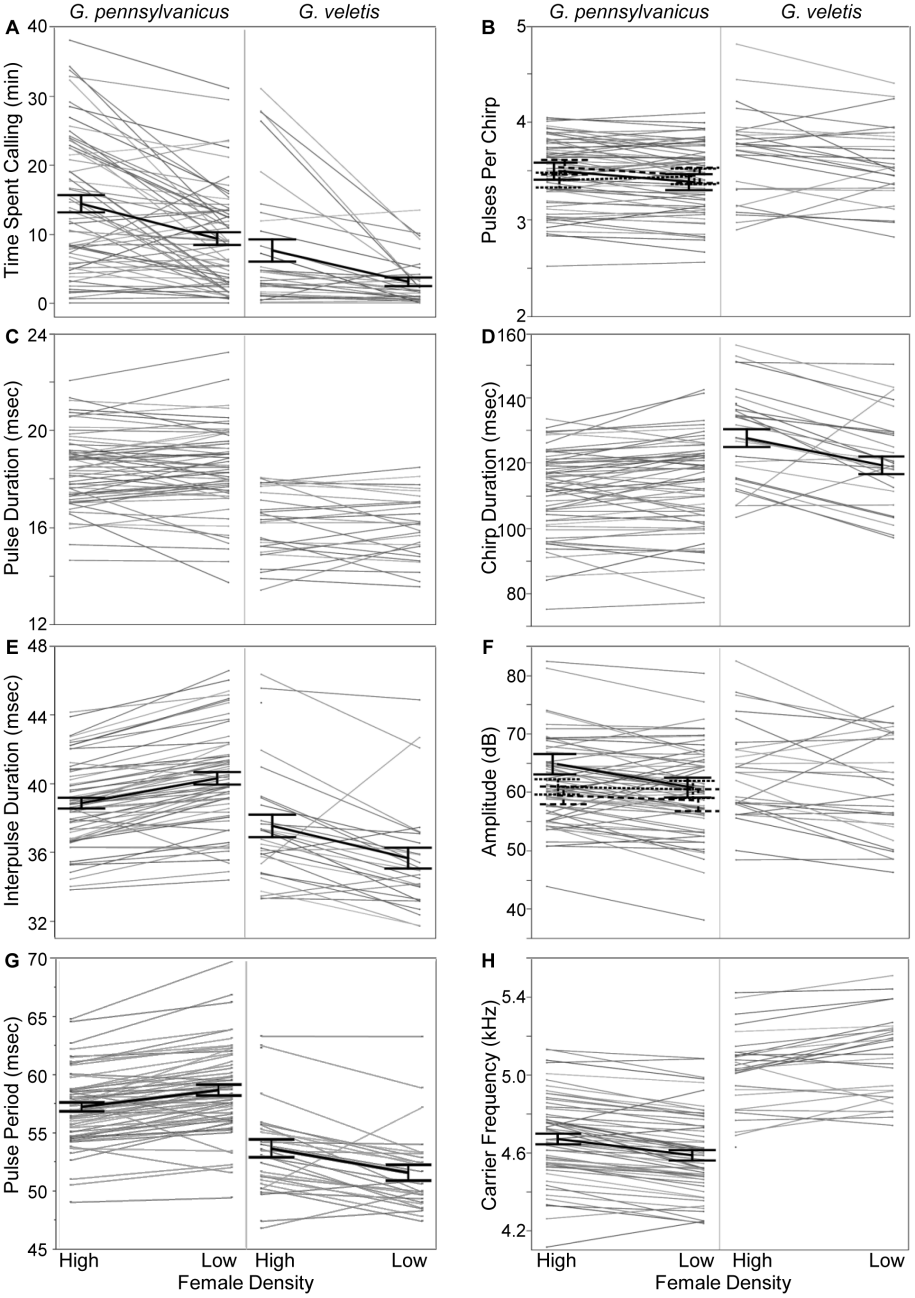
Signaling plasticity across times of high and low mating activity in nature. Mating activity in nature: *G. pennsylvanicus* high = 22∶00–09∶59, low = 10∶00–21∶59; *G. veletis* high = 04∶00–15∶59, low = 16∶00–03∶59 ([32]). Each thin gray line represents an individual. Significant time period effects are shown using heavy black lines; significant interactions between mating activity and size are shown with three lines (solid - large, dotted - medium, dashed - small); statistics are shown in Tables 2–4.

Male crickets used phenotypically plastic signals, generally investing more effort into long distance mate attraction signaling during the time of day that mating activity is highest in the wild. Our models investigating whether individuals exhibit plasticity in their signaling behavior were highly significant, explaining 71–97% of the variation in signaling traits (Tables 2–4). Time period, representing periods of high or low mating activity in the wild, explained 3–22% of the signaling variation; individual identity explained 54–93% (Tables 2–4). During times that mating activity is highest in the wild (22∶00–09∶59; [32]), *G. pennsylvanicus* males generally signaled more often, with shorter breaks between pulses and shorter pulse periods, more pulses per chirp, louder chirps, and higher carrier frequencies (Figures 1 and 3; Tables 2 and 3). Male *G. pennsylvanicus* were, however, highly variable in their signaling plasticity, with a few males signaling in a manner that appeared maladaptive. For example, some males signaled less often and with quieter signals when mating activity is typically highest and signaled more often and louder when mating activity is typically lowest. Body size explained some of these differences in phenotypic plasticity. However, contrary to our prediction, average-sized males did not exhibit more phenotypic plasticity in their amplitude or pulses per chirp across mating activity contexts; rather, large and small (based on pronotum area) males did. Large and small males signaled louder and with more pulses per chirp when mating activity is highest in the wild and quieter with fewer pulses per chirp when mating activity is lowest. Residual mass did not explain differences in phenotypic plasticity.

**Table 2:** How signaling effort plasticity is influenced by time of day (subdivided by mating period), residual mass, and body size.

Species	IndependentVariables	DF	F Ratio	P	R^2^_adj_	IV%
*G. pennsylvanicus*	Mating Activity(MA)	1,58	26.8220	**<0.0001**	0.8068	66.12
	Residual Mass(RM)	2,58	0.3436	0.7107		
	Body Size(BS)	2,58	1.0531	0.3554		
	RM * MA	2,58	1.4248	0.2489		
	BS * MA	2,58	1.1146	0.3350		
*G. veletis*	Mating Activity(MA)	1,26	11.7550	**0.0020**	0.7673	61.41
	Residual Mass(RM)	2,26	0.0556	0.9460		
	Body Size (BS)	2,26	1.4155	0.2609		
	RM * MA	2,26	0.0250	0.9753		
	BS * MA	2,26	0.9030	0.4177		

Repeated measures ANOVA model output investigating plasticity in signaling time across high and low mating activity time periods; MA = Mating Activity; RM = Residual Mass, BS = Body Size; IV% = Variance explained by individual effects in the repeated measures model; FDR_B–Y_ alpha P<0.0120. Note: this legend also applies to Table 3 and 4.

**Table 3:** How *G. pennsylvanicus* fine scale signaling parameter plasticity is influenced by time of day (subdivided by mating period), residual mass, and body size.

SignalingTraits	IndependentVariables	DF	F Ratio	P	R^2^_adj_	IV %
PulseDuration	Mating Activity (MA)	1,57	0.0124	0.9116	0.9205	84.85
	Residual Mass (RM)	2,57	1.3350	0.2713		
	Body Size (BS)	2,57	1.6897	0.1937		
	RM * MA	2,57	0.7575	0.4735		
	BS * MA	2,57	1.3054	0.2790		
InterpulseDuration	Mating Activity (MA)	1,57	95.5756	**<0.0001**	0.9535	90.76
	Residual Mass (RM)	2,57	0.4490	0.6405		
	Body Size (BS)	2,57	0.8869	0.4175		
	RM * MA	2,57	0.5570	0.5760		
	BS * MA	2,57	0.6657	0.5179		
PulsePeriod	Mating Activity (MA)	1,57	47.0348	**<0.0001**	0.9413	88.92
	Residual Mass (RM)	2,57	0.2271	0.7976		
	Body Size (BS)	2,57	0.6112	0.5462		
	RM * MA	2,57	0.8750	0.4224		
	BS * MA	2,57	0.0027	0.9973		
PulsesPer Chirp	Mating Activity (MA)	1,57	8.9179	**0.0042**	0.9614	92.85
	Residual Mass (RM)	2,57	0.4277	0.6541		
	Body Size (BS)	2,57	0.2076	0.8132		
	RM * MA	2,57	0.3012	0.7411		
	BS * MA	2,57	6.6881	**0.0025**		
ChirpDuration	Mating Activity (MA)	1,57	4.2342	0.0442	0.9522	91.06
	Residual Mass (RM)	2,57	0.8741	0.4228		
	Body Size (BS)	2,57	0.4781	0.6224		
	RM * MA	2,57	0.7047	0.4985		
	BS * MA	2,57	2.6607	0.0786		
InterchirpDuration	Mating Activity (MA)	1,57	0.1573	0.6931	0.8842	79.89
	Residual Mass (RM)	2,57	0.0832	0.9203		
	Body Size (BS)	2,57	0.9505	0.3926		
	RM * MA	2,57	0.6129	0.5453		
	BS * MA	2,57	1.7478	0.1834		
Amplitude	Mating Activity (MA)	1,57	13.5078	**0.0005**	0.9326	87.26
	Residual Mass (RM)	2,57	0.4173	0.6608		
	Body Size (BS)	2,57	1.3263	0.2735		
	RM * MA	2,57	0.6394	0.5314		
	BS * MA	2,57	5.5090	**0.0065**		
CarrierFrequency	Mating Activity (MA)	1,57	85.1932	**<0.0001**	0.9719	93.87
	Residual Mass (RM)	2,57	0.6229	0.5400		
	Body Size (BS)	2,57	4.4165	0.0165		
	RM * MA	2,57	4.1034	0.0216		
	BS * MA	2,57	1.7301	0.1865		

**Table 4:** How *G. veletis* fine scale signaling parameter plasticity is influenced by time of day (subdivided by mating period), residual mass, and body size.

SignalingTraits	IndependentVariables	DF	F Ratio	P	R^2^_adj_	IV %
PulseDuration	Mating Activity (MA)	1,22	0.6028	0.4457	0.8996	79.81
	Residual Mass (RM)	2,23	2.3163	0.1213		
	Body Size (BS)	2,23	2.5329	0.1015		
	RM * MA	2,22	0.7563	0.4812		
	BS * MA	2,22	2.5092	0.1043		
InterpulseDuration	Mating Activity (MA)	1,21	10.5859	**0.0037**	0.8439	73.45
	Residual Mass (RM)	2,22	0.0534	0.9481		
	Body Size (BS)	2,22	1.3417	0.2818		
	RM * MA	2,21	0.5118	0.6065		
	BS * MA	2,21	0.2086	0.8134		
PulsePeriod	Mating Activity (MA)	1,21	12.0577	**0.0022**	0.8845	80.79
	Residual Mass (RM)	2,22	0.4665	0.6332		
	Body Size (BS)	2,22	0.2223	0.8024		
	RM * MA	2,22	0.7880	0.4674		
	BS * MA	2,22	0.9810	0.3911		
PulsesPer Chirp	Mating Activity (MA)	1,22	2.3425	0.1400	0.8978	80.52
	Residual Mass (RM)	2,23	2.3490	0.1180		
	Body Size (BS)	2,23	0.5839	0.5658		
	RM * MA	2,22	0.3506	0.7081		
	BS * MA	2,22	1.2535	0.3050		
ChirpDuration	Mating Activity (MA)	1,22	12.3159	**0.0019**	0.8364	69.23
	Residual Mass (RM)	2,23	1.5760	0.2283		
	Body Size (BS)	2,23	0.3790	0.6887		
	RM * MA	2,22	0.3121	0.7351		
	BS * MA	2,22	2.7546	0.0853		
InterchirpDuration	Mating Activity (MA)	1,22	5.6811	0.0261	0.7803	66.20
	Residual Mass (RM)	2,23	0.1097	0.8965		
	Body Size (BS)	2,23	0.0798	0.9236		
	RM * MA	2,22	1.5068	0.2434		
	BS * MA	2,22	1.1061	0.3484		
Amplitude	Mating Activity (MA)	1,23	4.6936	0.0411	0.8104	58.79
	Residual Mass (RM)	2,23	3.4634	0.0483		
	Body Size (BS)	2,23	4.9375	0.0164		
	RM * MA	2,23	0.3832	0.6860		
	BS * MA	2,22	0.4849	0.6221		
CarrierFrequency	Mating Activity (MA)	1,22	5.2173	0.0326	0.7053	54.40
	Residual Mass (RM)	2,22	1.0355	0.3717		
	Body Size (BS)	2,22	0.5955	0.5599		
	RM * MA	2,22	0.1494	0.8621		
	BS * MA	2,22	0.3335	0.7201		

*Gryllus veletis* males also used phenotypically plastic signals and invested more effort into mate signaling during times that mating activity is highest in the wild (04∶00–15∶59; [32]). Males generally signaled more often, with longer breaks between pulses, pulse periods, and chirp durations when mating activity is typically highest in the wild (Figure 3; Tables 2 and 4). Similar to *G. pennsylvanicus*, *G. veletis* males were also highly variable in their plasticity, with a few signaling in an apparently maladaptive manner (Figure 3). Some males signaled less often and more quietly during times that mating activity is highest in the wild and more often and louder when mating activity is lowest. Neither body size nor residual mass significantly explained among-male variation in phenotypic plasticity.

## Discussion

Given that the density of conspecific rivals and potential mates determines the intensity of sexual selection [33]–[38], male field crickets should be selected to exhibit adaptive phenotypic plasticity in their mate attraction signals that matches the socio-sexual competitive context they experience. Our findings support this idea. Most males signaled plastically, aligning their mate attraction signals to the temporally fluctuating socio-sexual context they faced. Males signaled for mates following distinct diel rhythms that differed across species. Male signaling diel rhythms are synchronized so that they are in phase with female mating activity diel rhythms in the wild. Male *G. pennsylvanicus* signaled more often (14 versus 9 min/hr), louder (62 vs 60 dB), with elevated carrier frequencies (4.7 vs 4.6 kHz), shorter interpulse durations (38 vs 40 msec), and shorter pulse periods (57 vs 59 msec) during the high versus low mating activity time periods, respectively (high 22∶00–09∶59 vs low 10∶00–21∶59; [32]). This plasticity appears adaptive given that female *G. pennsylvanicus* preferentially mate with males that signal most often [63] and are more attracted to loud signals played at 5 kHz versus quiet ones played at 4 kHz, and signals with pulse periods falling within the 35–60 msec range [49], [54]. Male *G. veletis* signaled more often (7 vs 3 min/hr), with longer interpulse durations (37 vs 35 msec), longer pulse periods (53 vs 50 msec) and longer chirp durations (128 vs 118 msec) during the high versus low mating activity time periods (high 04∶00–15∶59 vs low 16∶00–03∶59; [32]). While less is known about female *G. veletis*’ mating preferences, one study revealed females are attracted to signals with pulse periods that ranged from 40–70 msec [54]. Female *G. veletis* may be similar to other cricket species and preferentially mate with males that signal most often and with long chirp durations [42], [44], [63]–[66]. If so, plasticity in male *G. veletis* signaling traits would be adaptive.

Species-level differences in temporal signaling rhythms also appear adaptive. The mating seasons of *G. pennsylvanicus* and *G. veletis* overlap spatially and temporally. Since *G. pennsylvanicus* and *G. veletis* both breed in grassy fields and rock crevices (SMB, SJH, IRT, LPF), it is likely that their habitats overlap in mid-summer. *Gryllus pennsylvanicus* and *G. veletis* differ in the time of day when peak calling and mating activity occurs, with *G. pennsylvanicus* peaking at night and *G. veletis* peaking in the morning. Given the fine-scale structure of most mating signal parameters overlap extensively across the two species, these temporal differences may act as a behavioral barrier to reproductively isolate the two species.

While we conclude that males are changing their signaling throughout the day in response to female mating activity, it is also possible that females are instead mating more during these times of day because males are signaling more. The causal relationships between male signaling and female mating activities should be explored by measuring female receptivity to mating throughout the day while controlling for male signaling behavior.

### Condition Dependency?

Sexually selected traits should act as phenotypically plastic gauges of an individual’s condition because only individuals in top condition should be able to bear the costs of advertising with the most exaggerated traits. Predictions differ about how condition influences plasticity. Bretman et al. [29] predicted a concave relationship between condition and plasticity, where males in high and low condition exhibit minimal plasticity, whereas males in intermediate condition exhibit high plasticity. They theorized that intermediate condition males should have the stores available to amplify their signals when environmental conditions are best, but not enough stores to constantly maintain amplified signals. Conversely, Jacot et al. [37] predicted a negative linear relationship between condition and plasticity. They suggested males in low condition should exhibit high phenotypic plasticity because they only have sufficient resources available to invest when the net benefits are highest. These differences in predictions likely results from how the authors define low condition males, but regardless, our results do not support either of them. Although variation in body size (pronotum area) explained some of the variation in signaling plasticity in *G. pennsylvanicus*, the largest individuals exhibited the highest adaptive plasticity, not the intermediate-sized individuals (as predicted by Bretman et al. [29]) or smallest individuals (as hypothesized by Jacot et al. [37]). Body size is partially indicative of resource availability during development [66], but is also a heritable trait in many field cricket species [67]–[70]. Larger individuals may have superior genotypes, making larger males capable of both growing larger during development and exhibiting more adaptive signaling plasticity during adulthood, a hypothesis that requires testing.

Residual mass did not explain variation in signaling plasticity. We assumed that males with low residual mass were in worse condition than males with average or high residual mass. However, some low residual mass males may be better at acquiring resources and therefore more willing to risk investing energy into signaling, because it can be easily replaced. If so, this would have confounded our results for how residual mass influenced plasticity. Our *ad libitum* feeding regime following field capture could also have confounded our results for how residual mass influences plasticity. For these reasons and many others [57], [71]–[74], residual mass may not be a strong measure of condition in crickets [75], [76].

Hill [23] recommends quantifying condition using physiological, cellular, and biochemical processes. Supporting Hill’s [23] assertion, our recent research suggests that differences in carbohydrate metabolism may more accurately denote condition in chirping crickets [29]. Metabolic power for signaling comes from work caused by the thoracic muscles closing the plectrum against the file during the production of a sound pulse [77]. Energetic signaling costs are dependent on the total number of pulses produced and on the average number of teeth struck during the production of a pulse [78]–[80]. While the energetic costs of chirping are not very high, reaching only 1–4× resting metabolic rate [78]–[85], the costs may be high enough for some males to not be able to alter the quality or quantity of their signals. Future research should examine whether plastic signaling is tied to the ability to metabolize carbohydrates.

### Temperature-Induced Plasticity?

Our fine scale signaling plasticity results were unlikely to result from minor changes in ambient temperature. We controlled for temperature by conducting our study in a climate-controlled greenhouse. However, while the temperature remained close to 28°C throughout our experiment, it occasionally climbed as high as 30°C during the day and fell as low as 26°C at night. Increasing temperature *decreases* interpulse, chirp and interchirp durations [78], [86]–[91]. The slight temperature fluctuations experienced in the greenhouse are unlikely to explain the signaling plasticity because we observed that interpulse, chirp, and interchirp durations *increased* throughout the morning hours, as the greenhouse warmed with the morning sun.

### Maladaptive Behavior?

A handful of males behaved in a seemingly maladaptive manner, consistently producing unattractive signals or signaling most during the time of day that mating activity is lowest in the wild. Why do some males signal maladaptively? While *G. pennsylvanicus* mating activity is highest at night and around sunrise (22∶00–09∶59), some mating (12%) occurred during the rest of the day (10∶00–21∶59; [32]). Similarly, while *G. veletis* mating activity is highest in the early morning and throughout the day (04∶00–15∶59), some mating (19%) occurred during evening hours (16∶00–03∶59; [32]). Males that signal more when mating activity is lowest may be less aggressive or less able to compete with males during peak mating hours, and may instead signal during times of lower competition. Variation in signaling plasticity could also stem from age, fighting experience, or mating experience differences among males. We captured males as adults in the field, so age and social experience are unknown. Male signaling changes with age (*G. pennsylvanicus*: [92]; *G. veletis*: [93]) and so males may also alter signaling plasticity with age/experience.

Male variation in signaling plasticity could also stem from female behavioral plasticity in the wild. Wild female *G. pennsylvanicus* and *G. veletis* are likely to exhibit variation in the types of signals to which they are most attracted. Whereas female field crickets are generally most responsive to specific signaling parameters, some females exhibit narrow mating preferences while other females exhibit broad preferences [54]. Female age and mating history may play a role, because older and mated females are typically much choosier than younger and virgin females [54]. Virgin females are more likely to mate and mate more quickly than previously-mated females [94]. Differences in choosiness could result from physiological differences, changes in hormonal levels altering auditory receptiveness, and/or lower residual reproductive value [95]. Female social experience could also shape differences in preference. The perceived attractiveness of previously encountered males influences pre- and post-copulatory mate choice decisions with subsequent males [96]. Given that females in the wild may exhibit mate preference plasticity, the seemingly maladaptive mate signaling strategies of males may in fact not be maladaptive.

Regardless of the underlying causes of some individuals signaling more attractively or more often during the non-peak mating hours, this signaling may be risky. Signaling during non-peak mating hours may increase the potential of interbreeding (*G. pennsylvanicus* with *G. veletis*). Given hybridization is lethal [43], signaling more attractively or more often during the non-peak mating hours may be maladaptive.

### Conclusions and Future Directions

Phenotypic plasticity can be adaptive when phenotypes align with environmental changes. In crickets, environmental changes result in rhythmic fluctuations in the density of sexually active individuals (summarized in Table S1). Given that density strongly influences the intensity of sexual selection [33]–[38], we asked whether crickets exhibit plasticity in their signaling behavior that aligns with these rhythmic fluctuations in the socio-sexual environment. Our findings suggest that most crickets exhibit phenotypically plastic mate attraction signals that match the temporally fluctuating socio-sexual context they experience. Our findings also suggest cricket signaling plasticity may be adaptive. Our correlational study exploring variation in plasticity is the first step towards determining whether crickets exhibit genotype by environment interactions in their signaling behaviors (e.g., [58]–[60]). Future research should (1) quantify the signaling plasticity of crickets reared in the laboratory to allow us to control for mating experience, condition, and age effects [97, submitted] and (2) examine whether plastic signaling is tied to the ability to metabolize carbohydrates. If signaling plasticity of crickets reared in the laboratory is similar to wild crickets, then future research should also (3) quantify the underlying genetic basis of signaling plasticity.

## Supporting Information

Table S1 **Literature review of how cricket and parasitoid density change over the course of a day.** Studies with different results are presented in separate rows, and studies with the same results are presented in the same row. A dash (−) indicates the species was not observed during that time period. ^a^ French Polynesia; ^b^ Australia; ^c^ Hawaii;.
(DOCX)

Table S2 **Repeated measures ANOVA using repeated measures general linear mixed models (GLMM) utilizing the restricted maximum likelihood (REML) approach to investigate whether signaling behavior changed over the course of a day.** Individual was classified as a random effect, while time was classified as a fixed effect using six time periods: Time: 1 = 00∶00–03∶59; 2 = 04∶00–07∶59; 3 = 08∶00–11∶59; 4 = 12∶00–15∶59; 5 = 16∶00–19∶59; 6 = 20∶00–23∶59. Separate models were run for *G. pennsylvanicus* and *G. veletis* and for each of the 9 signaling parameters. Our corrected alpha FDR_B-Y_ level of significance is P<0.0143 to account for the 18 models run. Our models were generally highly significant explaining 55–88% of the variation in signaling traits: time of day explained 3–15% of the signaling variation, while individual identity explained 40–85%.
(DOCX)
