# Supplementary material for: Adaptive Plasticity in Wild Field Cricket’s Acoustic Signaling
Source: PLoS One. 2013 Jul 23;8(7):e69247. doi: 10.1371/journal.pone.0069247 (PMC3720581; doi:10.1371/journal.pone.0069247)
Supplement: Table S1 — Literature review of how cricket and parasitoid density change over the course of a day. Studies with different results are presented in separate rows, and studies with the same results are presented in the same row. A dash (−) indicates the species was not observed during that time period. a French Polynesia; b Australia; c Hawaii;. (DOCX) [file pone.0069247.s001.docx]

| **Type** | **Species** | **Sex** | **Behavior** | 00:00-1:59 | 02:00-03:59 | 04:00-05:59 | 06:00-07:59 | 08:00-09:59 | 10:00-11:59 | 12:00-13:39 | 14:00-15:59 | 16:00-17:59 | 18:00-19:59 | 20:00-21:59 | 22:00-23:59 | **Study** | **Study** |
| --- | --- | --- | --- | --- | --- | --- | --- | --- | --- | --- | --- | --- | --- | --- | --- | --- | --- |
| Tachinid | *Homotrixa alleni* | Female | Attraction | L | L | L | - | - | - | - | - | L | L | H | H | Field | ^98^ |
| Bush Cricket | *Sciarasaga quadrata* | Male | Signaling | L | L |  |  |  |  |  |  | L | M | H | H | Field | ^98^ |
|  |  | Female | Mating | - | - | - | - | - | - | - | - | - | - | - | - |  | ^98^ |
| Tachinid | *Ormia ochracea* | Female | Attraction | L | L | L |  |  |  |  |  |  |  | H | H |  | ^99^ |
| Field Cricket | *Gryllus texensis* | Male | Signaling | M | H | H | H | H | - | - | - | - | M | M | M | Field | ^31^ |
|  |  | Male | Signaling | M | M | M | H | M | M | M | M | M | M | M | M | Field | ^32^ |
|  |  | Female | Mating | L | L | H | H | H | M | M | L | L | L | L | L |  | ^32^ |
| House Cricket | *Gryllodes sigillatus* | Male | Signaling | L |  | M | H | H | H | M | H |  |  |  | L | Lab | ^100^ |
|  |  | Female | Mating | L | L | M | M | M | M | M | M | L | L | L | L | Lab | ^100^ |
| Field Cricket | *Gryllus veletis* | Male | Signaling | M | M | H | H | H | H | H | L |  |  | L | M | Field | ^32^ |
|  |  | Female | Mating | L | L | H | H | H | M | M | L | L | L | L | L | Field | ^32^ |
|  |  | Male | Signaling | M | L | L | M | H | H | M | H | L | H | H | M | Lab | ^100^ |
|  |  | Female | Mating | H | H | M | L | L | L | L | M | H | H | H | H | Lab | ^100^ |
|  |  | Male | Signaling | H | H | H | M | M | L | L | L | L | M | M | H | Field | ^101^ |
| Field Cricket | *Gryllus campestris* | Male | Signaling | H | M | M | L | M | M | M | L | M | H | H | H | Field | ^102^ |
|  |  | Female | Mating |  |  |  | L | L | M | H | M | H | M | M | L | Field | ^102^ |
| Field Cricket | *Gryllus pennsylvanicus* | Male | Signaling | M | H | H | H | M | M | L | L | L | M | M | M | Field | ^32^ |
|  |  | Female | Mating | H | H | H | H |  |  |  |  |  |  |  | H | Field | ^32^ |
| Field Cricket | *Gryllus campestris* | Male | Signaling | L | L | L | L | L | M | M | H | H | H | H | H | Field | ^37^ |
|  |  | Female | Mating | L | L | L | L | L | L | M | H | H | H | H | L | Field | ^37^ |
| Field Cricket | *Gryllus supplicans* | Male | Signaling |  |  |  | L | M | H | H | H | M |  |  |  | Lab | ^103^ |
| Tachinid | *Ormia ochracea* | Female | Attraction | L |  |  |  |  |  |  |  |  | H | H | M | Field | ^104^ |
| Field Cricket | *Teleogryllus oceanicus* | Male | Signaling | H | H | H | H |  |  |  |  |  | L | M | H | Lab | ^104^ |
|  |  | Male | Signaling | L | L | L |  |  |  |  |  |  | H | H | L | Field^c^ | ^104^ |
|  |  | Male | Signaling | H | H | H |  |  |  |  |  |  | L | H | H | Lab | ^105^ |
|  |  | Female | Walking | H | H | H | H | M | L |  |  |  |  | H | H | Lab | ^105^ |
|  |  | Male | Signaling | H | H | H | L |  |  |  |  | L | L | H | H | Field | ^105^ |
|  |  | Male | Signaling | H | H | H | L |  |  |  |  | L | H | H | H | Field^a^: | ^106^ |
|  |  | Male | Signaling | H | H | H | L |  |  |  |  | L | H | H | H | Field^b^ | ^106^ |
|  |  | Male | Signaling | H | H | H | L |  |  |  |  |  | L | H | H | Field^c^ | ^106^ |
| Field Cricket | *Gryllodes supplicans* | Male | Signaling | H | H | M | - | - | - | - | - | - | - | H | H | Field | ^107^ |
| Mole Cricket | *Scapteriscus vicinus* | Male | Signaling |  |  |  |  |  |  |  |  |  |  | H |  | Field | ^108, 109^ |
| Mole Cricket | *Scapteriscus ocieturs* | Male | Signaling |  |  |  |  |  |  |  |  |  |  | H |  | Field | ^108, 109^ |
| Shorttailed Cricket | *Anurogryllus arboreus* | Male | Signaling |  |  |  |  |  |  |  |  |  | L | H | L | Field | ^108, 110, 111^ |
| Shorttailed Cricket | *Anurogryllus muticus* | Male | Signaling | H | M | M |  |  |  |  |  |  | L | H | H | Field | ^108, 112^ |
