# Supplementary material for: Adaptive Plasticity in Wild Field Cricket’s Acoustic Signaling
Source: PLoS One. 2013 Jul 23;8(7):e69247. doi: 10.1371/journal.pone.0069247 (PMC3720581; doi:10.1371/journal.pone.0069247)
Supplement: Table S2 — Repeated measures ANOVA using repeated measures general linear mixed models (GLMM) utilizing the restricted maximum likelihood (REML) approach to investigate whether signaling behavior changed over the course of a day. Individual was classified as a random effect, while time was classified as a fixed effect using six time periods: Time: 1 = 00∶00–03∶59; 2 = 04∶00–07∶59; 3 = 08∶00–11∶59; 4 = 12∶00–15∶59; 5 = 16∶00–19∶59; 6 = 20∶00–23∶59. Separate models were run for G. pennsylvanicus and G. veletis and for each of the 9 signaling parameters. Our corrected alpha FDRB-Y level of significance is P<0.0143 to account for the 18 models run. Our models were generally highly significant explaining 55–88% of the variation in signaling traits: time of day explained 3–15% of the signaling variation, while individual identity explained 40–85%. (DOCX) [file pone.0069247.s002.docx]

|  | **Signaling Parameter** | **DF** | **F Ratio** | **P** | **R^2^_adj_** | **ID Var%** |
| --- | --- | --- | --- | --- | --- | --- |
| *G. pennsylvanicus* | Time Spent Calling | 5,310 | 14.5000 | **<0.0001** | 0.5976 | 49.13 |
|  | Pulse Duration | 5,287.6 | 1.0449 | 0.3914 | 0.7873 | 74.47 |
|  | Interpulse Duration | 5,287.7 | 40.7797 | **<0.0001** | 0.7742 | 68.78 |
|  | Pulse Period | 5,287.7 | 33.8852 | **<0.0001** | 0.7950 | 72.63 |
|  | Pulses Per Chirp | 5,287.4 | 2.2000 | 0.0544 | 0.7922 | 75.29 |
|  | Chirp Duration | 5,287.3 | 22.8353 | **<0.0001** | 0.8282 | 78.59 |
|  | Interchirp Duration | 5,286.70 | 11.2159 | **<0.0001** | 0.7247 | 67.28 |
|  | Amplitude | 5,287.7 | 3.8170 | **0.0023** | 0.7609 | 70.99 |
|  | Carrier Frequency | 5,287.4 | 28.4454 | **<0.0001** | 0.8836 | 85.01 |
| *G. veletis* | Time Spent Calling | 5,155 | 7.5680 | **<0.0001** | 0.5645 | 45.28 |
|  | Pulse Duration | 5,100.9 | 1.7254 | 0.1355 | 0.7273 | 67.93 |
|  | Interpulse Duration | 5,100.4 | 15.3564 | **<0.0001** | 0.7064 | 58.74 |
|  | Pulse Period | 5,100.20 | 14.9851 | **<0.0001** | 0.7153 | 60.22 |
|  | Pulses Per Chirp | 5,101.50 | 8.5771 | **<0.0001** | 0.7753 | 70.24 |
|  | Chirp Duration | 5,101 | 31.0613 | **<0.0001** | 0.8086 | 70.33 |
|  | Interchirp Duration | 5,103.50 | 7.8255 | **<0.0001** | 0.6099 | 44.55 |
|  | Amplitude | 5,102.60 | 1.8689 | 0.1062 | 0.5515 | 58.04 |
|  | Carrier Frequency | 5,102.00 | 4.2642 | **0.0015** | 0.6233 | 52.48 |
